# Editorial Comment: Oncological and functional outcomes of open versus laparoscopic partial nephrectomy in T1b tumors: A single-center analysis

**DOI:** 10.1590/s1677-5538.ibju.2018.0865.1

**Published:** 2020-02-20

**Authors:** Luciano A. Favorito

**Affiliations:** 1 Universidade do Estado de Rio de Janeiro - Uerj Unidade de Pesquisa Urogenital Rio de JaneiroRJ Brasil Professor Associado da Unidade de Pesquisa Urogenital - Universidade do Estado de Rio de Janeiro - Uerj, Rio de Janeiro, RJ, Brasil; 2 Hospital Federal da Lagoa Serviço de Urologia Rio de JaneiroRJ Brasil Serviço de Urologia, Hospital Federal da Lagoa, Rio de Janeiro, RJ, Brasil

## COMMENT

Kartal and Collegues from Turkey in this important paper studied the oncological and functional results of open partial nephrectomy (OPN) and laparoscopic partial nephrectomy (LPN) at the T1b clinical stage in 63 patients and compared 41 submitted to OPN and 22 submitted to LPN (1). The authors observed that there are no differences between OPN and LPN techniques between oncological and functional outcomes in patients with clinical stage T1b RCC.

Partial nephrectomy (open, laparoscopic or robotic) is considered the gold standard for treating localized renal tumors (2-6). In last years important technical improvements were introduced with robotic surgery. Recently a interesting study suggested that the robotic technique is a valid option for partial nephrectomy, especially for PADUA<10 lesions but without differences between surgical techniques in more complex masses (7). The present paper confirms previous findings (8-10) that open and laparoscopic surgery still has indications in complex cases and (because the robotic costs) in countries in development.
